# Life in Extreme Conditions: Diet and Condition of the Extremophile Fish *Aphanius almiriensis* (Teleostei: Cyprinodontiformes) in a Thermal Rheocrene Spring

**DOI:** 10.1002/ece3.71411

**Published:** 2025-05-07

**Authors:** Gülşah Saç, Oya Özuluğ, Sevan Ağdamar, Harun İnci, Özgün Deniz Yürekli, Müfit Özuluğ

**Affiliations:** ^1^ Department of Biology, Faculty of Science Istanbul University İstanbul Türkiye; ^2^ Department of Forestry, Bayramiç Vocational School Çanakkale Onsekiz Mart University Çanakkale Türkiye; ^3^ Institute of Science, Department of Biology Istanbul University İstanbul Türkiye

**Keywords:** benthic insect, biotic interactions, extreme habitat, feeding ecology, killifish

## Abstract

This study aims to understand the bioecological traits of an extremophile fish, *Aphanius almiriensis*, in order to explore how it survives and colonizes extreme habitat conditions. To achieve this, the bioecological characteristics—condition, diet, and feeding strategy—of *A. almiriensis* inhabiting the Tuzla thermal spring, which is characterized by extreme habitat conditions in terms of physicochemical water parameters, were studied. Among the physicochemical parameters measured, salinity and temperature were remarkably high, ranging from 23.7°C (in winter) to 42.7°C (in summer) and from 47.7 ppt (in autumn) to 60.7 ppt (in winter). A total of 248 fish individuals were collected from the thermal spring, and the diet analyzed seasonally consisted of 17 different food items, and their importance values (MI% and IRI%) varied seasonally. According to the F%, IRI%, and MI% values, the population fed mainly on Diatom, Cyanobacteria, and Diptera, resulting in niche breadth with low values ranging from 0.25 to 0.54. The extreme conditions of the thermal spring indicated that the environment was poor in terms of macroinvertebrate diversity (six taxa), and fish showed food selectivity (E) for Chironomidae and Ceratopogonidae in winter, spring, and summer and for Ephydridae in autumn (E > 0; positive selectivity). Seasonally influenced fish condition was represented by high values in summer (K = 1.43 ± 0.14) and was positively correlated with both water temperature and the increasing importance of the genus *Phormidium* in its diet. This study provides the first comprehensive insight into the seasonal diet and condition of *A. almiriensis*, shedding light on its survival strategies in harsh ecological conditions.

## Graphical abstract

This study aims to answer some questions about the bioecological traits of an extremophile fish *Aphanius almiriensis* in order to understand how it can survive and colonize the extreme habitat conditions. To this end, some bioecological characteristics—condition, diet, and feeding strategy—of *A. almiriensis* inhabiting the Tuzla thermal spring, which has extreme habitat conditions in terms of physicochemical water parameters, were studied.

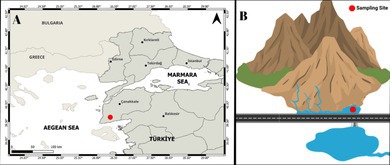

## 1 Introduction

Both hypersaline and thermal waters are important extreme habitats on the planet because the interaction of different abiotic parameters makes these environments less hospitable to living organisms and creates a particular trophic structure that limits species richness (Akbarzadeh et al. 2014; Shadrin and Anufriieva 2020; Kornyychuk et al. 2023). Animals constantly interact with both biotic and abiotic factors in their environment to survive and meet their basic needs. Abiotic factors such as water temperature, salinity, pH, and DO have a significant impact on the health, survival, and growth of organisms, as well as their productivity and sustainability (Ding et al. 2022; Menon et al. 2023). While abiotic environmental factors can cause stress by exceeding tolerance thresholds or becoming lethal, biotic factors, especially food, are crucial for the survival of most animals (Shadrin and Anufriieva 2020). Many species have developed homeostatic mechanisms that help them cope with stress when environmental conditions temporarily exceed their tolerance limits (Dove et al. 2005). However, feeding is of vital importance, and the availability of resources, especially under extreme habitat conditions, affects the diet of animals, and individuals may survive depending on resource utilization.

Extremophiles are organisms that thrive in environments considered inhospitable to most other organisms due to the presence of physicochemical stressors, and they often develop complex adaptations to cope with these stressors (Plath et al. 2015). Killifishes are recognized as members of the extremophilic group of fishes, found in diverse habitats, including tropical and temperate, freshwater, and estuarine environments, ranging from desert ecosystems to thermal springs and hypersaline waters, and encompassing more than 1200 species (Akbarzadeh et al. 2014; Chiozzi et al. 2018; Piller et al. 2022). These species can cope with large spatiotemporal variations in environmental parameters and survive through their ability to adapt to different variations in temperature and salinity (Cavraro et al. 2017). In addition to their tolerance to high temperature and salinity, they can also tolerate varying degrees of organic and inorganic pollution and low oxygen levels (Bakhtiyari et al. 2011). These different environmental conditions lead these fish to develop different feeding behaviors, and their diet varies according to the availability of food, ranging from animals (such as zooplankton, aquatic insects, crustaceans, fish, and mollusks) to plant material (such as diatoms, filamentous algae, and leaves) and detritus in different habitat types (Alcaraz and García‐Berthou 2007; Bakhtiyari et al. 2011; Güçlü and Küçük 2008; Leonardos 2008; Yoğurtçuoğlu et al. 2018).

The killifishes of the genus *Aphanius* are small fishes of marine origin from the Mediterranean basin. While *Aphanius fasciatus* has a wide distribution in the basin, *Aphanius almiriensis* was described from the Aegean Basin (Peloponnese, Greece) in 2007 and very limited populations were listed up to date (Kottelat et al. 2007; Triantafyllidis et al. 2007; Valdesalici et al. 2019). Populations of *A. almiriensis* range from estuaries to coastal lagoons in both Greece and Türkiye and the species has recently been recorded from a small coastal cenote in Italy, outside its known range (Kottelat et al. 2007; Valdesalici et al. 2019). Although the killifish has been known for many years in the Tuzla geothermal area in the northwest Aegean (Çanakkale, Türkiye) and was thought to be *A. fasciatus* , a molecular and morphological study recently identified it as *A. almiriensis* (Ağdamar et al. 2024; Freyhof and Yoğurtçuoğlu 2020). The habitat conditions of this population are quite remarkable; it inhabits a field where geothermal waters and brines originate from the Tuzla cliffs in a tectonically active region and form small shallow springs or pools.

The feeding ecology of fish is closely linked to population dynamics, including fertility, growth, condition, and maintenance (McKay et al. 2022), and population sustainability can be supported by appropriate feeding behavior. Therefore, the study of feeding ecology is crucial for understanding the relationships between organisms and food resources in an ecosystem, such as prey selection, predation, as well as evolution, competition, and energy transfer within these habitats (Braga et al. 2012). Extreme habitat conditions with limited biotic diversity and food supply require fish to develop feeding behaviors that are specifically adapted to these environments. However, changes in the availability of food resources or seasonal variations in prey populations can influence the feeding ecology of fish. As a result, fish develop adaptive feeding behaviors, and this strategy allows them to adapt quickly to fluctuations in food abundance or availability and to secure food intake in fluctuating environments (Laske et al. 2018).

This study aims to answer the following questions about the bio‐ecological traits of *A. almiriensis* to understand how it can survive and colonize the extreme conditions of the Tuzla thermal rheocrene spring: (a) What is the diet and niche breadth of *A. almiriensis* under the extreme habitat conditions of the spring? (b) Are there any temporal differences in the feeding of the fish? (c) What is the prey selectivity of the fish in its extreme habitat and how has it changed seasonally? and (d) What is the relationship between the feeding strategy and the condition of the fish under extreme environmental variables?

## 2 Methods

**FIGURE 1:**
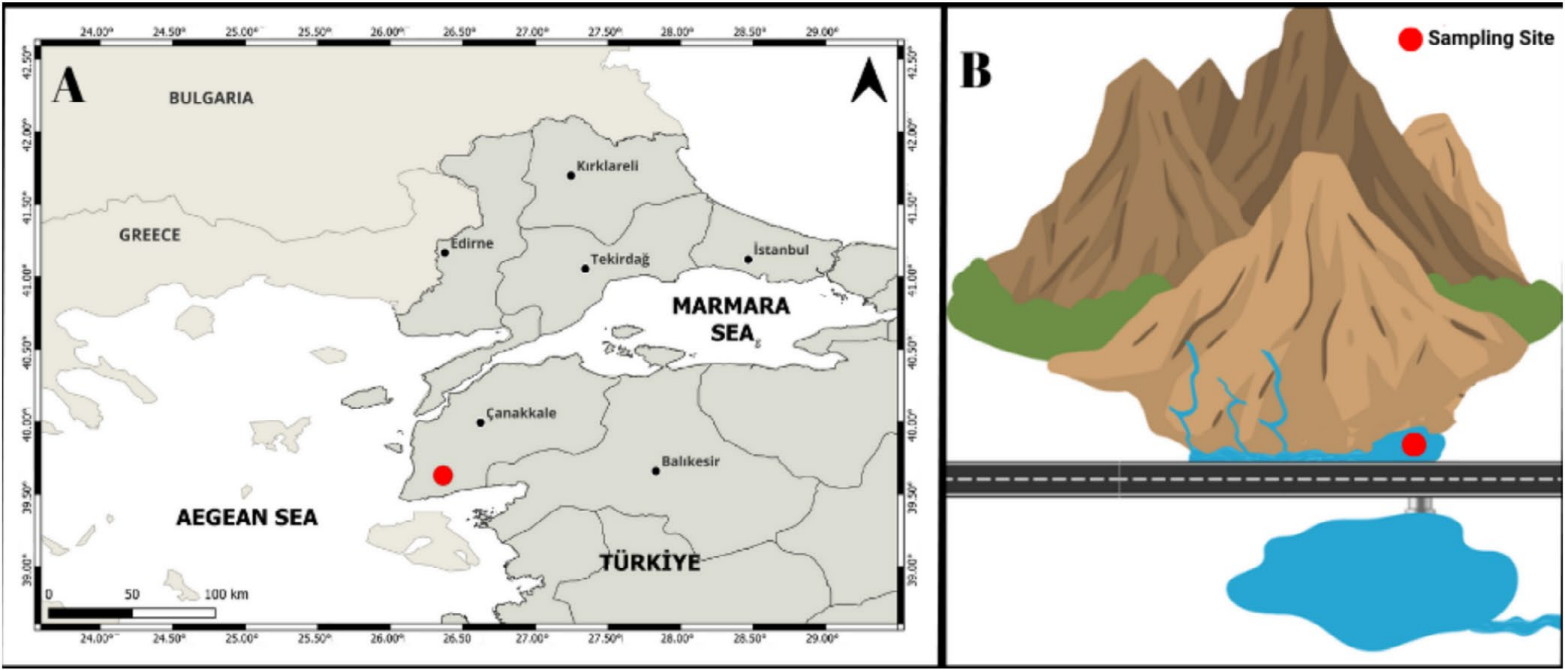
Study area at Tuzla thermal spring, Çanakkale, Türkiye. (A) The location of the study area in Türkiye and (B) the study area.

**FIGURE 2:**
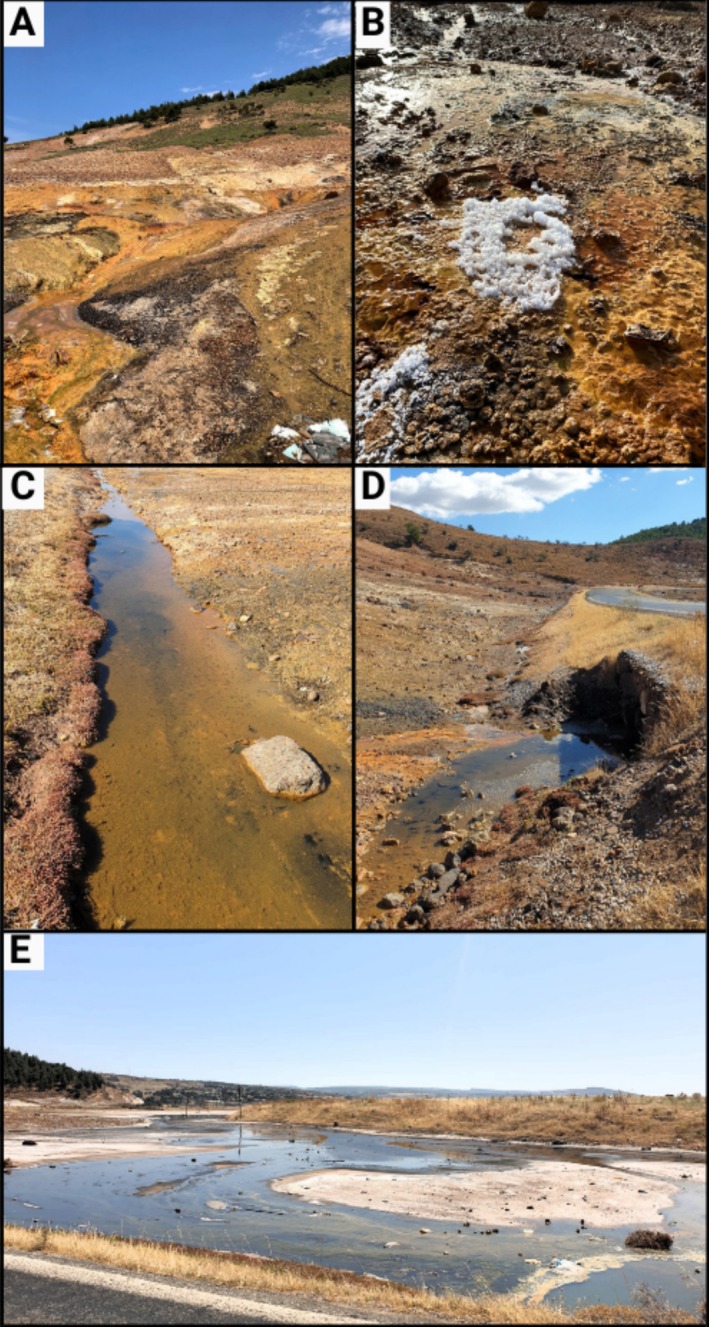
The Tuzla thermal rheocrene spring, Çanakkale, Türkiye. (A) Water discharging from the rock, (B) salt formations, (C) water flowing to a small puddle, (D) small puddle consisting of flowing cold and hot water, and (E) marsh area of the spring.

The study area is located in the foothills of Tuzla Mountain (Çanakkale, Türkiye) in a region with thermal water outlets. Two independent thermal rheocrene springs were observed by the study team in the region, and this study was carried out in only one of these. Tuzla thermal rheocrene spring (39.57°69′50″ N 26.16°91′61″ E) is a small marshy pool (about 20 m in diameter) at the foot of Tuzla Mountain and is fed both by trickling water at a temperature of about 70°C, which is discharged by geothermal evaporation, and by cold leakage water in the rock (Figures 1, 2). The first place where these waters flowing through the rocks mix is a small puddle, 1 m in diameter and about 30 cm deep, which spreads out and becomes shallow, forming a marshy area. It then flows through a small canal into the Tuzla stream, which has a connection to the northeastern Aegean Sea. Although the spring flows throughout the year, its volume is reduced by evaporation during the summer months, and in some places, it dries up until the autumn rains arrive. Figure 1 was created using QGIS (ver. 3.32 Lima) and www.canva.com.

Field surveys were conducted seasonally (once per season, from January to October 2023) at the Tuzla thermal spring. Fish were collected from the small puddle at the sampling site (Figures 1, 2) using a 3 mm mesh scoop, with each sampling taking an average of 15 min. As the water was shallow, the fish were easily visible, allowing one person to guide them into the scoop while the other collected them with a scoop. After being captured, the fish were placed in buckets containing an overdose of clove oil (100 mg/L) and ethically euthanized in accordance with the animal welfare regulations of the Republic of Türkiye. Water temperature (WT, °C), salinity (S, ppt), dissolved oxygen (DO, mg L^−1^), conductivity (EC, μS cm^−1^), and pH were evaluated as abiotic environmental variables. These physicochemical properties of the water were measured using a multiparameter (Eutech CyberScan 600). Aquatic macroinvertebrates were sampled as a biotic environmental factor, as they may serve as food for fish and interact with them. Aquatic macroinvertebrates were sampled within a 1 m^2^ quadrat area using a special hand net with a mesh size of 0.5 mm. Both the fish and the macroinvertebrates were transferred to the laboratory in a formalin solution (1%).

Fish were measured to the nearest 0.1 cm for total length (TL) and 0.01 g for body mass. Sex was determined by sexual dimorphism in the coloration of the flanks, as described by Kottelat et al. (2007). A Chi‐square test was used to compare the observed sex ratio (female to male) with the theoretical 1:1 ratio (Zar 1999). Condition was calculated using Fulton's condition factor *K* = (*W*/*L* ^3^) × 100, where W represents total mass (g) and L represents total length (cm) (Ricker 1975). Normal distribution (Kolmogorov–Smirnov) was first checked before comparing condition factor (*K*) values. Accordingly, seasonal differences in condition factor values were tested using Kruskal–Wallis. To describe the magnitude of the difference between parameters, the Eta‐squared (*η* ^2^) was determined, and the post hoc test (Dunn test) was performed (R Core Team 2022; Rdocumentation 2025).

To determine diet composition, the digestive tracts were removed from fish samples, and food items were categorized to the lowest possible taxonomic level using both binocular and microscope. After identification, countable food items, such as insect groups, were counted, and then all types of food were dried at 80°C (2–4 h) and weighed (to the nearest 0.0001 g). The macroinvertebrates, both in the environment and in the digestive tract of the fish samples, were identified using specific identification keys (Askew 1988; Bouchard Jr. 2012; Nilsson 1996, 1997; Oscoz et al. 2011; Zwick 2004).

The proportion of full stomachs (PFS%) was calculated to examine the feeding intensity of the population. To assess the level of consumption of specific food items, population feeding habits were measured by (a) relative importance index (IRI) for macroinvertebrates, and (b) modified relative importance index (MI) for all food items.

The IRI was applied specifically to macroinvertebrates due to their ease of counting. However, the MI was calculated separately for all food items, considering that certain items, such as plant debris or *Phormidium*, are difficult to quantify, while others like diatoms or cyanobacteria might lead to miscounts due to their large numbers. The two indices were calculated as follows: (a) IRI = (*N*% + *W*%) × *F*% and (b) MI% = *F*% × *W*%. F% represents the percentage frequency of occurrence [(number of digestive tracts containing a food item/total number of digestive tracts with food) × 100], *N*% is the numerical percentage of digestive tracts with a certain food item against the total number of digestive tracts, and *W*% is the gravimetric percentage of a certain food item against the weight of all consumed taxa (Hyslop 1980).

Using Levin's standardized index (Hurlbert 1978), the dietary niche breadth at the population level was assessed as follows: *B*_*A*_ = (*B*–1)/(*n*–1), where B is Levins' measure of niche breadth (Levins 1968) and *n* is the number of food items. The standardized index provides a measure of niche breadth on a scale from 0 to 1. *B*_*A*_ values close to 0 indicate more narrowly focused (minimum niche breadth) and specialized diets, and *B*_*A*_ values higher than 0.6 indicate broader (the population consumes the available food items in equal proportions) and more generalized diets and the values of *B*_*A*_ are considered intermediate when between 0.4 and 0.6 and low when below 0.4 (Novakowski et al. 2008).

Macroinvertebrates in the environment were identified and the individuals of each taxon were counted to determine the *N*_*e*_% (relative abundance of each food item in the environment) using the following equation: *N*_*e*_% = number of individuals of a taxon (*N*_*e*_)/total number of individuals of all taxa (total *N*_*e*_) × 100 (Kocataş 2008). To assess the prey preferences of the fish on the macroinvertebrates, Ivlev's electivity index (*E*) was calculated. The index is calculated as follows: *E* = (*r*_*i*_−*P*_*i*_)/(*r*_*i*_ + *P*_*i*_), where *E* = electivity index, *r*_*i*_ = percentage of food item *i* in the diet (*N*_*f*_%), and *P*_*i*_ = percentage of food item *i* in the environment (*N*_*e*_%). The value of *E* ranges from −1 to +1, and it indicates a positive selectivity if it is greater than 0 and a negative selectivity when it is less than 0. Values equal to or greater than 0.6 are considered high selectivity (Pinto and Uieda 2007).

## 3 Results

The physicochemical parameters of the sampling area are listed in Table 1 on a seasonal basis. In the extreme conditions of the Tuzla thermal spring, water temperature and salinity were particularly high, ranging from 23.7°C (in winter) to 42.7°C (in summer) and from 47.7 ppt (in autumn) to 60.7 ppt (in winter), respectively. Dissolved oxygen decreased with increasing water temperature in summer, while TDS showed a higher value (83.92 ppt, spring) compared to other seasons, especially in parallel with spring precipitation.

**TABLE 1:** Seasonal variation of physicochemical properties of the water in the Tuzla thermal spring (Çanakkale, Türkiye).

Parameter	Winter	Spring	Summer	Autumn
Salinity (S, ppt)	60.7	59.8	55.2	47.7
Water temperature (WT, °C)	23.7	37.1	42.7	40.0
Dissolved oxygen (DO, mg L^−1^)	8.94	7.19	5.51	7.49
Conductivity (EC, μS cm^−1^)	76.3	79.9	75.0	63.9
pH	6.99	6.95	6.62	6.81

A total of 248 fish individuals were collected from the Tuzla thermal spring, and their total length (TL, cm) and weight (W, g) distributions varied between 1.8–4.2 cm and 0.06–1.04 g, respectively (Table 2). Average K (± SD) values for female, male, and all individuals were 1.18 (± 0.21), 1.15 (± 0.22), and 1.18 (± 0.21), respectively. Seasonal variation in K was calculated for both sexes and all individuals, and the results showed that K increased to maximum values in summer for both sexes (Table 2). As a result of the Kolmogorov–Smirnov test, it was determined that the data set was not normally distributed (*p* < 0.05), and the Kruskal–Wallis selected for significance found a significant difference between the seasons (*p* < 0.05). According to the Eta‐Squared result, the significant difference between seasons is high (η^2^ = 0.643). According to the results of the post hoc test (Dunn test), the largest significant difference was found between summer and winter (*p* < 0.05). Although there was a significant difference between autumn–summer, spring–summer, and autumn–winter (*p* < 0.05), there was no significant difference between autumn–spring and spring–winter (*p* > 0.05). The sex ratio of females to males was found to be 4.4:1.0, which is significantly different from the ratio of 1:1 (*x* ^*2*^ = 54.45; *p* < 0.05).

**TABLE 2:** The sexual and seasonal ranges of individual numbers (*n*), total length (TL, cm), body mass (W, g), and condition factor (K) values of *Aphanius almiriensis* from Tuzla thermal spring (Çanakkale, Türkiye).

Seasons	Sex	*n*	TL, cm (min.–max.)	W, g (min.–max.)	K (min.–max.)
Winter	♀	54	2.1–3.5	0.11–0.47	0.84–1.22
	♂	12	2.6–3.2	0.15–0.30	0.84–1.12
Spring	♀	33	2.1–3.8	0.10–0.64	0.76–1.44
	♂	7	2.4–3.0	0.14–0.28	0.91–1.12
Summer	♀	66	2.1–4.2	0.10–1.04	1.05–1.77
	♂	16	2.0–3.2	0.11–0.39	1.10–1.80
Autumn	♀	49	1.8–3.6	0.06–0.50	0.87–1.26
	♂	11	2.3–2.8	0.13–0.22	0.95–1.18
Total		248	1.8–4.2	0.06–1.04	0.76–1.80

The percentage of full stomachs (%) had the highest values for each season, ranging from 97.6% in summer to 100.0% in spring and autumn. The diet of the fish was categorized into 17 food items, ranging from cyanobacteria to fish (Table 3). Aquatic insects consisted of Diptera, Coleoptera, unidentified body parts, and eggs, while identifiable diatoms were *Navicula*, *Nitschia*, and *Halamphora*. In addition, the presence of the same fish species in the digestive tracts of only a few individuals indicates cannibalism, albeit at a low level (1.2%).

**TABLE 3:** Seasonal values of frequency of occurrence (F%), relative importance index (IRI%), the modified index of relative importance (MI%), proportion of full stomachs (PFS%), number of food items (n), and Levins' standardized index (B_A_) values for the food items consumed by the *Aphanius almiriensis* population in the Tuzla thermal spring (Çanakkale, Türkiye).

Food items	Winter			Spring			Summer			Autumn		
	F%	IRI%	MI%	F%	IRI%	MI%	F%	IRI%	MI%	F%	IRI%	MI%
Insecta*	18.5	24.0	1.6	27.5	1.9	1.5	3.8	0.6	< 0.1	3.3	1.1	< 0.1
Diptera*	1.5	0.5	0.1	5.0	0.2	0.3	10.0	4.8	0.1	1.7	0.3	< 0.1
Diptera–Chironomidae	3.1	0.7	< 0.1	25.0	2.8	1.6	6.3	2.6	< 0.1	5.0	3.2	< 0.1
Diptera–Ceratopogonidae	15.4	29.9	1.6	57.5	90.5	41.4	35.0	91.6	0.7	28.3	86.0	1.0
Diptera–Ephydridae	24.6	44.6	2.8	35.0	4.5	4.0	2.5	0.4	< 0.1	8.3	9.5	0.5
Coleoptera	1.5	0.2	< 0.1	5.0	0.1	0.1	2.5	0.3	< 0.1	0	0	0
Insect eggs	3.1	—	< 0.1	40.0	—	2.8	6.3	—	< 0.1	36.7	—	1.6
Arachnida **	1.5	—	< 0.1	2.5	—	< 0.1	0	—	0	0	—	0
Acaridae**	1.5	—	< 0.1	0	—	0	0	—	0	0	—	0
Nematoda	6.2	—	0.2	27.5	—	1.3	2.5	—	< 0.1	3.3	—	< 0.1
Annelida	1.5	0.2	< 0.1	0	0	0	0	0	0	0	0	0
Fish	1.5	—	0.1	0	—	0	2.5	—	< 0.1	0	—	0
Plant debris	40.0	—	7.0	12.5	—	0.3	12.5	—	22.5	16.7	—	0.4
Pollen	0	—	0	80.0	—	11.3	16.3	—	0.1	11.7	—	0.2
Diatom	100	—	43.9	100	—	17.6	100	—	38.2	100	—	12.0
*Phormidium*	0	—	0	12.5	—	0.3	98.8	—	22.5	93.3	—	73.6
Coccoid cyanobacteria	98.5	—	42.6	100	—	17.6	100	—	38.2	95.0	—	10.8
PFS (%)	98.5			100			97.6			100		
*n*	15			14			14			12		
B_A_	0.25			0.54			0.31			0.40		

*Note:* * Indicates unidentified taxa and ** indicates terrestrial forms.

**FIGURE 3:**
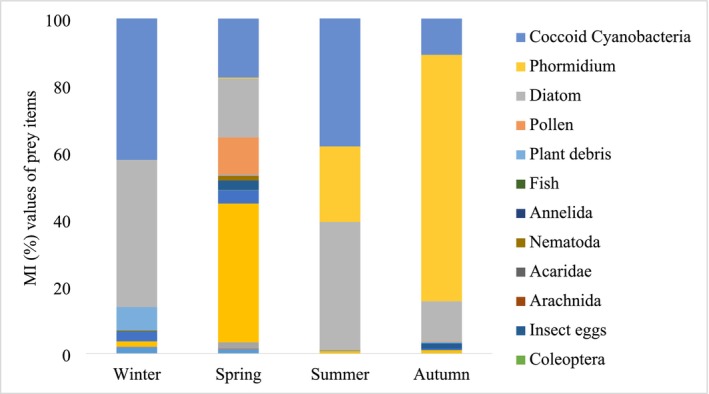
MI (%) values of prey taxa in the stomach contents of *Aphanius almiriensis* (*n* = 248) sampled at the Tuzla thermal spring. Dietary metrics for all prey taxa are presented in Table 3.

Food diversity is high across all seasons (Table 3, Figure 3), although the importance index values (MI%) for many food items remain relatively low, the population primarily feeds on diatoms, cyanobacteria, and dipterans. Diatoms were consumed by all fish in all seasons, closely followed by coccoid cyanobacteria, and they were represented with high importance values (MI%) compared to other food items in almost all seasons. The genus *Phormidium*, which was rarely found in fish stomachs in spring, dominated with a very high value in terms of importance index (MI%) in autumn. The insects consumed were mainly Diptera, and rarely Coleoptera, with Ceratopogonidae being the most consumed dipteran in all seasons. Among the identifiable insect groups, Ephydridae had high importance values (both MI and IRI) in winter, while Ceratopogonidae was the dominant insect in all other seasons. Plant debris was found in the digestive tract of each individual in all seasons, but the frequency of occurrence of plant debris was highest in winter, while the importance value increased in summer. Arachnida and Acaridae individuals found in the diet were terrestrial forms and were rarely observed.

Niche breadth (B_A_) was estimated to be 0.33 for all individuals examined over the entire study period. The seasonally estimated B_A_ values are shown in Table 3. Despite the number of food types (*n* = 14) detected for spring, the B_A_ value was found to be the highest for this season.

A total of 124 macroinvertebrate samples of six identified taxa were collected during the study period, as well as *A. almiriensis*, and the proportion of samples of each taxon by season was given (Table 3). The samplings in the study area indicate that this extreme habitat is poor in terms of macroinvertebrate diversity: Ephydridae were sampled from the spring in all seasons, but Annelidae and Hirudinea only in winter. Although Chironomidae and Ceratopogonidae were not sampled in winter and spring, they were consumed by fish in all seasons. This is an indication that they are indeed present in the environment and selected by fish. Coleoptera was sampled from the environment only in spring, but it was consumed by the fish in all seasons except autumn.

The fish were found to be positively selective for Chironomidae, Ceratopogonidae, and Annelidae according to the total result of the study. However, although the fish consumed Ephydridae or Coleoptera, they were not selective for them. The negative selectivity for these groups can be explained by the higher abundance of these prey in the environment than in the fish diet. Seasonal analysis shows that although three different macroinvertebrate groups were sampled from the Tuzla thermal spring in winter, the fish consumed only Ephydridae individuals and were negatively selective for them (Table 4). Although chironomids, coleopterans, and annelids were not obtained in the environment, they were consumed by the fish in low numbers, resulting in positive selectivity. Only Coleoptera and Ephydridae were sampled in spring, and although the fish consumed them as food, they did not show positive selectivity. In summer and autumn sampling, the environment was represented by only three different groups of Diptera, while the fish were positively selective for Chironomidae and Ceratopogonidae in summer and for Ephydridae in autumn.

**TABLE 4:** Electivity index (E) values calculated for *Aphanius almiriensis* living in the Tuzla thermal spring (Çanakkale, Türkiye) during the sampling seasons (r_i_ = percentage of food item *i* in the diet of fish, N_e_% = percentage of food item *i* in the environment).

Taxa	Winter			Spring			Summer			Autumn			Total		
	r_i_ (%)	N_e_%	E	r_i_ (%)	N_e_%	E	r_i_ (%)	N_e_%	E	r_i_ (%)	N_e_%	E	r_i_ (%)	N_e_%	E
Chironomidae	5.0	—	1	5.5	—	1	12.7	11.1	0.08	13.8	20.8	−0.20	6.6	4.8	0.15
Ceratopogonidae	47.5	—	1	90.1	—	1	79.4	77.8	0.03	65.5	70.8	−0.04	85.0	19.4	0.63
Ephydridae	42.5	93.3	−0.37	4.0	90.2	−0.91	4.8	11.1	−0.39	20.7	8.3	0.43	7.4	69.4	−0.81
Coleoptera	2.5	—	1	0.4	9.8	−0.92	3.2	—	1	—	—	—	0.8	4.8	−0.71
Annelidae	2.5	—	1	—	—		—	—	—	—	—	—	0.2	0	1
Hirudinea	—	3.3	−1	—	—		—	—	—	—	—	—	0	0.8	−1
Gastropoda	—	17.6	−1	—	—		—	—	—	—	—	—	0	0.8	−1

## 4 Discussion

This is the first study on the diet and condition of *A. almiriensis*, newly described from some extreme habitats in the Mediterranean basin, so there is no other study with which to compare and interpret the results. However, previous studies on *A. fasciatus*, the other species of the genus, which also occurs in the Mediterranean basin, may help us to evaluate or compare our results. It should be noted that the area studied is a habitat with extreme conditions, and fish living in such a habitat have to develop a unique life and feeding strategy. Firstly, like other killifishes (Alcaraz et al. 2015; Alcaraz and García‐Berthou 2007; Esmaeili and Shiva 2006), the population structure of *A. almiriensis* exhibited a distinct sex ratio, with females being more abundant than males (4.4:1.0). According to Esmaeili and Shiva (2006), the biased sex ratio reflects the differences in survival rate between sexes and the investment in females is manifested in higher survival rates and longer life spans of females, or their greater resilience to environmental stressors. Sex ratio is a key factor in population demography and the evolution of breeding systems, affecting both population viability and biodiversity conservation (Székely et al. 2014), and a female‐biased strategy may also be effective in the persistence of *A. almiriensis* populations in its extreme habitat conditions. Secondly, the Tuzla thermal spring is a small water body that is shallow and does not allow the development of aquatic plants. It is thought that these extreme conditions, together with the high water temperature or salinity, may also limit the diet of the fish. Before we started the study, it was an important question for us to understand how the fish could feed and survive in such a habitat. However, the fullness of the digestive tracts of the fish in each season has indicated that there was a seasonal continuity in the feeding activity of *A. almiriensis* and that all individuals were always able to feed in these extreme conditions. Similar to our results, Alcaraz et al. (2015) found that *A. farsicus* (valid as *Esmaeilius persicus*) feeds all year round due to the warm climate of the mesohaline Barm‐e‐Shoor spring in the Maharlu Lake Basin (Shiraz, Iran). Fish are poikilothermic organisms, and increased water temperature, through its effects on physiology and behavior, triggers the fish's ability and desire to feed (Cautant 1987; Volkoff and Rønnestad 2020). Temperature has also been recognized as a crucial factor in the biology of killifishes, through its effect on feeding activity (Alcaraz et al. 2015). It is therefore believed that the warm water of the Tuzla thermal spring allows *A. almiriensis* to feed opportunistically on the food available in the environment throughout the year.

*Aphanius almiriensis* is an omnivorous fish that consumes both plant‐ and animal‐origin food items, but it primarily feeds on unicellular organisms such as diatoms and coccoid cyanobacteria. In addition, its diet is characterized by the consumption of different groups, from the genus *Phormidium* and pollen to Ceratopogonidae and insect eggs, depending on the temporal variation in the food supply of the environment in terms of number and abundance. Although the availability of food in the environment is important in the temporal variation of fish feeding, whether their feeding patterns are passive (hang and wait) or active (move and hunt) (Saikia 2016), fish generally tend to feed on easier prey with less energy expenditure. In addition, diatoms are enriched with highly bioactive sources such as lipids, sterols, hydrocarbons, phenolics, polysaccharides, and alkaloids, making them the main food of the fish (Singh et al. 2022; Yi et al. 2017). Therefore, the fish fed intensively throughout the year on diatoms or cyanobacteria, which are particularly easy to collect and eat, grazing on them rather than feeding on insects or other mobile prey. The presence of sediment fragments, such as sand, in the digestive tracts, observed during microscopic examination, further confirmed that the fish had ingested diatoms while collecting them, indicating a predominantly grazing diet.

Inadequate food supply in the environment is also thought to determine the feeding behavior of fish. Thermal springs, which are also extreme habitats for the vital activities of many macroinvertebrate groups that can be consumed as food, can be considered a poor nutritional environment that limits the diet of fish. Such thermal springs are limited in terms of producers or consumers because they consist of abiotic factors that exceed or strain the tolerance limits of organisms (Benvenuto et al. 2015; Collins et al. 1976; İnci et al. 2024). As a matter of fact, during the sampling of the study area, a limited number of macroinvertebrate samples representing only six taxa, three of which are dipteran taxa with high ecological tolerance, were collected.

Diptera, which can survive and colonize nearly all freshwater habitats, from microthin trickles to waterfalls, and from glacial meltwaters to hot springs, is the most abundant and diverse group in aquatic environments, more so than any other group of macroorganisms (Adler and Courtney 2019; Sundermann et al. 2007). Due to their accessibility and abundance, they provide an important ecological service in the food web, particularly for fish (Courtney and Duffield 2000; Davies 1981). In the current study, three dipteran groups were relatively more abundant both in the environment and in the fish diet, in contrast to other macroinvertebrates. Each of the three dipteran groups, Ephydridae, Ceratopogonidae, and Chironomidae, has the ability to survive in the stressful habitats of thermal springs, which have high temperatures and salty water (Borkent and Spinelli 2007; Krivosheina 2003; Mullen et al. 1988; Torrejon et al. 2022). Ephydridae, which are known to be the dominant members of the macroinvertebrate fauna in extreme habitats such as saline and hot water springs (Brock et al. 1969; Foote 1995; Wirth 1971), were found both in the fish diet and the environment, but the selectivity of the fish gave positive values for the other two dipterans. It is thought that the fact that Chironomidae and Ceratopogonidae were not obtained from the environment but consumed by the fish during the winter and spring seasons contributed to this finding.

Since temperature, photoperiod, and salinity can alter production and consumption cycles and food availability, environmental conditions can influence the type of food and prey in the ecosystem (Bakhtiyari et al. 2011). Therefore, the diets or feeding strategies of ecologically tolerant killifishes differ depending on the environments from estuaries to small streams, from hypersaline waters in salt marshes to lakes, in which they live (Alcaraz et al. 2015; Alcaraz and García‐Berthou 2007; Leonardos 2008; Yoğurtçuoğlu et al. 2018). In the study of the diet of *A. fasciatus* in the Messolongi lagoon system (western Greece), Leonardos (2008) reported that the population (up to 6 cm total length) showed a seasonal and ontogenetic change in its diet, with smaller fish feeding on planktonic prey, mostly diatoms, and larger fish preferring benthic prey. Similarly, Yoğurtçuoğlu et al. (2018) reported that the diet of juveniles of the species *Anatolichthys marassantensis* was mostly based on planktonic organisms, while adults tended toward larger prey taxa. Since the length distribution of the fish collected in this study was in a narrow range (1.8–4.2 cm, TL), the ontogenetic differences in the diet of the fish were not analyzed. However, similar to the study by Leonardos (2008), it was found that, besides the diatoms, the fish generally hunted small prey, and about 90% of the macroinvertebrates (Diptera and Coleoptera only) they consumed were larvae. Considering the limited number of individuals larger than 3.5 cm that feed on the fish and drifting organisms (such as arthropods or adult insects), it is believed that if larger and more numerous individuals were studied, they could possibly consume larger prey found in the environment, such as gastropods. This could even result in cannibalism at a certain level. As a result, such larger prey may either become part of the fish's diet or become more important within it.

The diversity of food items and their importance in the diet indicated that the species may have a flexible diet depending on the environmental food supply. However, despite consuming a variety of food, the dominant feeding on some food items such as diatom, cyanobacteria, and genus *Phormidium* resulted in a low niche breadth. The B_A_ value for all individuals was estimated to be 0.33 with a narrow niche breadth, which is considered relatively low for the fish that appear to consume a total of 17 different food items. It is also thought that the dominance of some food groups listed above affects the diet diversity value being represented by low values such as niche breadth.

Killifishes are small‐sized fish, rarely reaching a total length of 8 cm, and average lengths in many populations are reported to be in the range of 4–5 cm (Leonardos 2008; Wildekamp et al. 1999; Yoğurtçuoğlu and Ekmekçi 2014, 2017). The total length range of *A. almiriensis* in the Tuzla thermal spring was 1.8–4.2 cm, with a mean length of 2.7 ± 0.44 cm, suggesting a dwarf population. Dwarf fish are characterized by maturing earlier and at a smaller size, and also tend to have a much shorter lifespan than normally growing fish (Trudel et al. 2001). It is a well‐known intraspecific rule that water temperature affects the metabolic rate, and in particular, individuals reared at higher temperatures develop faster and mature earlier but reach smaller adult body sizes (Lindmark et al. 2022; Ohlberger et al. 2011). In addition, many researchers have studied the effect of salinity on fish growth and have demonstrated that fish grow better at lower salinities, despite wide ranges of tolerance; this is because high or fluctuating salinities force fish to use more energy for osmotic and ionic regulation, leaving less energy for growth and development (Bœuf and Payan 2001; Kujawa and Piech 2022). Therefore, this hypersaline and thermal spring, where water temperatures and salinities as high as around 40°C and 50–60 ppt were detected, suggests that this population is effective in the development of this population as a dwarf population.

The seasonality of the condition factor may be caused by different breeding and feeding activities in different seasons, as well as environmental variables. In particular, in cold water conditions, fish condition values are generally low due to both the reduced food supply from the environment and the fact that they spend their energy developing their gonads rather than growing them. In summer, as the water temperature is optimal for growth and the greatest feeding activity begins, the condition of the fish increases (De Giosa et al. 2014; Dinh et al. 2022). The results of this study also showed that the condition of *A. almiriensis* was the lowest in winter, increased in spring when gonad development occurred, and reached its highest values in summer. Both the feeding strategy of the fish and environmental variables are effective on the condition of the fish. Seasonal variations in light levels may differentially affect species' foraging efficiency, or seasonal variations in temperature and precipitation may differentially affect species' metabolic rates (Bloomfield et al. 2022). Consequently, species may consume different food sources at different times of the year due to factors such as food availability, changes in energy obtained from their diet, or seasonal declines in feeding rates (Makri et al. 2024). *A almiriensis* consumed diatoms and cyanobacteria as food in all seasons of the year, but it was concluded that the significant importance of the genus *Phormidium* in the fish's diet, especially with increasing water temperature, had a positive effect on its condition.

In conclusion, this study is the first to investigate the condition and diet of *A. almiriensis*, offering valuable insight into how the species survives in extreme conditions by examining some of its bioecological properties. The diet of this omnivorous species consisted of 17 different food items; however, the species showed a narrow niche breadth as its main diet consisted of diatoms, cyanobacteria, and dipterans. While these three main food groups were consumed with high importance in all seasons, the feeding patterns of the species in some groups, such as the genus *Phormidium* and pollen, varied temporally according to the food supply in the environment. The limited food available to the fish under the extreme conditions of the thermal spring resulted in the species being selective in all seasons for macroinvertebrates, especially Chironomidae and Ceratopogonidae. It is believed that seasonally influenced conditions were related to both water temperature and feeding shifts to the genus *Phormidium*. The adaptation of the fish to this shallow area, which is open to environmental disturbance, as well as the physicochemical characteristics of the water, such as high temperature and salinity, is thought to be related to its efficient use of food in the environment and investment in females for the persistence of the population. In addition, this fish can be evaluated as a regional biological control agent against biting dipterans adapted to hypersaline waters and considered a threat to humans.

## Author Contributions

**Gülşah Saç:** conceptualization (lead), data curation (lead), formal analysis (lead), investigation (lead), methodology (lead), resources (lead), supervision (lead), writing – original draft (lead), writing – review and editing (lead). **Oya Özuluğ:** formal analysis (supporting), investigation (supporting), methodology (supporting). **Sevan Ağdamar:** data curation (supporting), formal analysis (supporting), funding acquisition (lead), investigation (supporting), project administration (lead), resources (lead), software (equal), writing – original draft (supporting), writing – review and editing (supporting). **Harun İnci:** formal analysis (supporting), investigation (supporting), software (supporting), visualization (supporting), writing – original draft (supporting). **Özgün Deniz Yürekli:** formal analysis (supporting), investigation (supporting), visualization (supporting). **Müfit Özuluğ:** formal analysis (supporting), investigation (supporting), writing – original draft (supporting), writing – review and editing (equal).

## Ethics Statement

This study was conducted with the approval of Çanakkale Onsekiz Mart University Committee of Animal Experiments Local Ethics (Decision ID: 2022/05–03).

## Consent

The authors have nothing to report.

## Conflicts of Interest

The authors declare no conflicts of interest.

## Data Availability

All data generated or analyzed during this study are included in this paper.
